# Transcriptional networks in whole blood of asthmatics

**DOI:** 10.1186/1710-1492-10-S2-A58

**Published:** 2014-12-18

**Authors:** Young Woong Kim, Amrit Singh, Casey P Shannon, Gail M Gauvreau, Scott J Tebbutt

**Affiliations:** 1 Experimental Medicine, University of British Columbia, Vancouver, British Columbia, V5Z 1M9, Canada; 2 James Hogg Research Centre, St. Paul’s Hospital, Vancouver, British Columbia, V6Z 1Y6, Canada; 3 Prevention of Organ Failure (ROOF) Centre of Excellence, Vancouver, British Columbia, V6Z 2K5, Canada; 4 Department of Medicine, McMaster University, Hamilton, Ontario, L8S 4L8, Canada

## Background

Allergen inhalation challenge causes significant changes in the blood transcriptomes of mild atopic asthmatic individuals [1]. Systems biology approaches have been used to identify transcriptional networks that reflect underlying disease processes. Such networks provide insights into the correlation patterns among genes and may identify specific molecular processes associated with asthmatic responses. Transcriptional networks can be identified using blood expression data and are associated with allergen-induced asthmatic responses.

## Methods

14 participants (8 early responders and 6 dual responders) with mild, atopic asthma underwent a cat allergen inhalation challenge as part of the AllerGen Clinical Investigator Collaborative. The subjects’ whole blood samples were collected immediately prior to allergen challenge (pre-challenge) and 2 hours after the challenge (post-challenge). Whole blood transcriptional profiling was performed using Affymetrix GeneChip ^®^ Human Gene 1.0 ST Arrays. The correlation networks (modules) were identified using weighted gene correlation network analysis (WGCNA) [2]. Pathway analysis was performed using GeneGo.

## Results

21,727 mRNA transcripts were profiled across 28 samples (14 pre and 14 post). Highly expressed mRNA transcripts (10,044, mean expression > log (base2) 6) were retained for WGCNA. WGCNA identified nine modules many of which were associated with various immune cell-types. A gene module consisting of 384 genes was significantly (p=0.0008) associated with the late phase asthmatic response and also significantly (p=1e-07) correlated with the compositional abundance of T cells. Pathways analysis of these genes indicated T cell receptor signaling pathway, TCR and CD28 co-stimulation in activation of NF-kβ and ICOS pathway in T-helper cells as the top significant pathways (FDR=10%).

## Conclusion

Transcriptional networks can be identified using whole blood expression data. Many transcriptional networks were associated with various cell-types frequencies, which may indicate the role of cell-specific gene expression in the development of asthmatic responses. Validation of these transcriptional networks will be performed using a larger asthma blood expression dataset.
